# Sinonasal Renal Cell-Like Adenocarcinoma: A Report of a Rare Entity With Emphasis on Its Association With Von Hippel Lindau Syndrome

**DOI:** 10.7759/cureus.38321

**Published:** 2023-04-30

**Authors:** Nasser M AlMadan, Abdulrahman Almohammed, Mahmoud Bardisi, Doaa AlGhamdi

**Affiliations:** 1 Dentistry, Prince Sultan Military Medical City, Riyadh, SAU; 2 Pathology, College of Medicine, Imam Muhammad Ibn Saud Islamic University, Riyadh, SAU; 3 Pathology, Riyadh Regional Lab and Blood Bank, Riyadh, SAU; 4 Pathology, King Fahad Medical City, Riyadh, SAU

**Keywords:** von hippel lindau disease, adenocarcinoma, sinonasal, snrcla, sinonasal renal cell like adenocarcinoma

## Abstract

Sinonasal renal cell-like adenocarcinoma (SNRCLA) is a rare malignant sinonasal tumor with relatively indolent clinical course. Clinically, it could be asymptomatic or show non-specific symptoms such as epistaxis, nasal obstruction, or hyposmia. Diagnosis of the lesion is challenging, especially in small biopsies, and requires clinical, radiological, histopathological, and ancillary tests to characterize the lesion accurately. We herein report a case of a 41-year-old female with a nasal mass noted two years ago, which presented initially as frequent epistaxis from the right side. Histopathological examination revealed proliferation of clear cells associated with hemorrhagic background forming follicular and glandular structure and dense eosinophilic secretion. Tumor cells were diffusely positive for CK7, EMA, and inhibin, while they were negative for CK20, P63, CK 5/6, CD10, renal cell carcinoma (RCC), TTF1, PAX8, CEA, and GATA3. The proliferation index (KI67) was less than 5%. The diagnosis was consistent with SNRCLA. The patient has no recurrence and no symptoms after one year. Thus, our study reports a rare case of SNRCLA with a discussion of the histological features and its association with von Hippel Lindau syndrome.

## Introduction

Sinonasal renal cell-like adenocarcinoma (SNRCLA) is a rare malignant tumor with around 20 cases reported in the literature [1,2]. The term “sinonasal renal cell-like adenocarcinoma” was described initially in two independent papers [3,4]. Under microscopy, it shows uniform low-grade clear tumor cells in a hemorrhagic background, with cells usually arranged in a follicular or glandular pattern. Tumor cells are crystal clear or contain fine eosinophilic material [5]. SNRCLA is usually managed with surgical resection, with some cases treated with adjuvant radiotherapy. It tends to have a good prognosis with rare recurrence [1]. One interesting aspect of the lesions is their association with von Hippel Lindau (VHL) syndrome, which was reported in three cases [2]. We herein present the first reported case of SNRCLA in Saudi Arabia with the morphological and immunohistochemical results and discuss its association with VHL syndrome.

This article was previously posted to the Research Square preprint server on February 9, 2023.

## Case presentation

**Figure 1:**
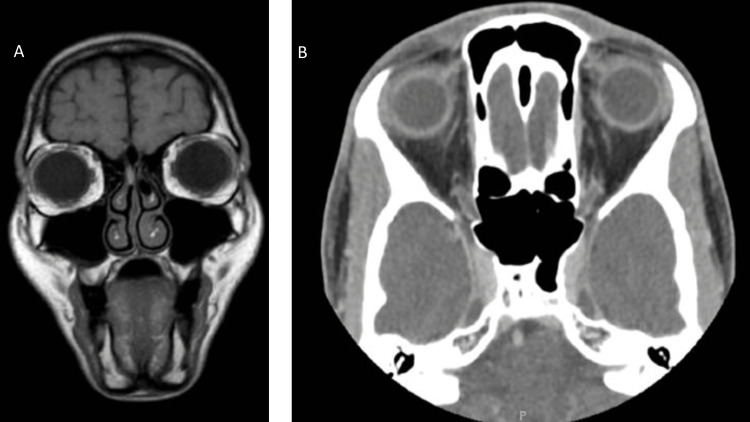
Radiographic image of the lesion MRI T1 coronal view (A) and CT scan axial view (B) showed a hyper-intense lesion in the right olfactory recess and right nasal cavity roof associated with thinning of the right cribriform plate with no involvement of the paranasal sinuses.

A 41-year-old female was referred to our hospital for a nasal mass with a preliminary diagnosis of olfactory neuroblastoma. The mass was noticed two years ago as frequent epistaxis from the right side. The patient had no hyposmia, nasal obstruction, facial numbness, or visual disturbance. The scope was performed, which showed a right bloody mass in the anterior skull base and congested turbinate. CT scan was performed, which showed opacification of the right olfactory recess and right nasal cavity roof with a low-density space-occupying lesion associated with thinning of the right cribriform plate with no involvement of the paranasal sinuses (Figure 1).

Positron emission tomography (PET) scan showed no uptake in any other organs, and whole body CT scan showed no other suspicious lesion. The patient underwent intranasal resection of the sinonasal tumor and anterior skull base and repair of skull base defect with fascia lata graft and lumbar drain insertion.

**Figure 2:**
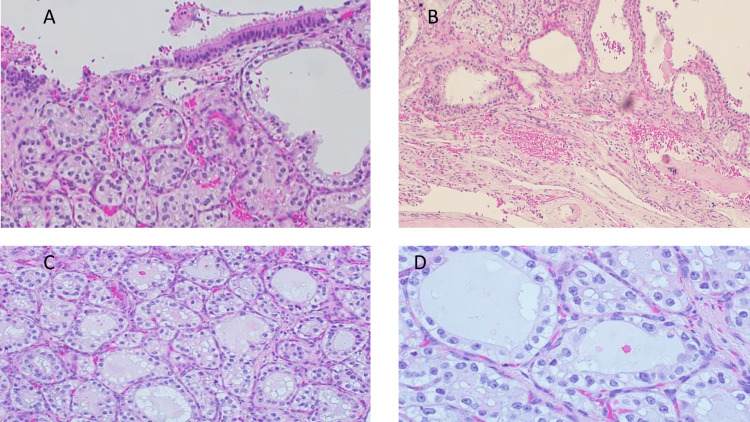
Histological features of the lesion (A) Tumor cell proliferation just below the respiratory lining and consisting of tumor cells with cuboidal clear cytoplasm and round nuclei with open chromatin and inconspicuous nucleoli with a lot of hemorrhage. (B) Tumor cells have nested and follicular arrangement with eosinophilic secretion in a hemorrhagic background. (C) Cells are arranged in back-to-back distribution with clear to eosinophilic secretion. (D) Higher power shows some cells with prominent nucleoli.

Histopathological examination revealed a minimally invasive tumor to the surrounding fibrous stroma with clear cells associated with hemorrhagic background forming follicular and glandular structure and dense eosinophilic secretion. Tumor cells showed cuboidal cells with rounded nuclei, fine chromatin, and some prominent nucleoli. Mitosis was seen, but it was rare, less than 1 per 2 mm^2^. No necrosis, bone, perineural, or perivascular invasion was identified (Figure 2).

**Figure 3:**
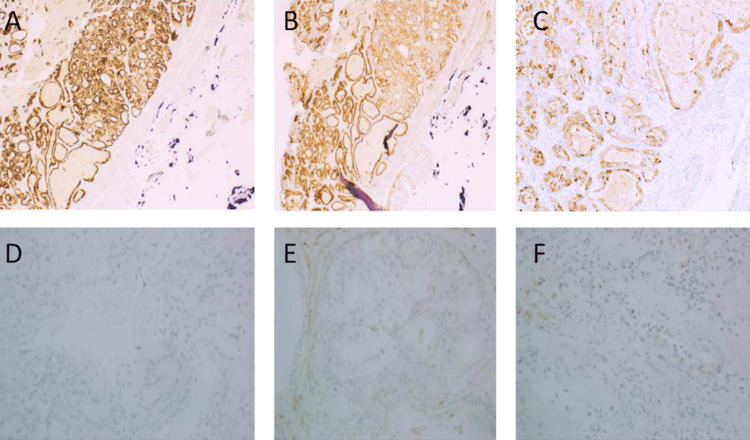
Ancillary tests Tumor cells are diffusely positive with CK7 (A) and EMA (B) and focally with inhibin (C), while they are negative for renal cell markers PAX8 (D), CD10 (E), and RCC (F).

Tumor cells were PAS-positive diastase labile. Additionally, it was diffusely positive for CK7, EMA, and inhibin, while they were negative for CK20, P63, CK5/6, CD10, RCC, TTF1, PAX8, CEA, and GATA3. The proliferation index (KI67) was less than 5% (Figure 3). The diagnosis was consistent with SNRCLA.

The patient has no tumor recurrence and no symptoms after one year with no other identified lesions with follow-up.

## Discussion

SNRCLA is a rare malignant sinonasal tumor that is thought to originate from sinonasal seromucinous glands [1,2,5]. The lesion was first described in 2002 by Zur et al. and Moh'd Hadi et al. in two independent papers [3,4]. The lesion has a slight female predilection with a mean age of 54 years (range: 22-89 years). Clinically, it could be asymptomatic or associated with non-specific symptoms such as epistaxis, nasal obstruction, hyposmia, or nasal discharge [1]. Radiographically, lesions could show expansile, locally destructive masses with intracranial extension and dural involvement. On MRI, the tumor is heterogeneous to the cortex on T2-weighted image and enhanced avidly, similar to RCC; therefore, it is essential to rule out the presence of a renal mass that metastasize into the sinonasal mucosa, which is far more common than SNRCLA [6].

Microscopically, it shows uniform low-grade clear tumor cells in a hemorrhagic background. The cells are usually arranged in a follicular or glandular pattern, with some lesions showing solid growth and focal micropapillary patterns. Tumor cells are crystal clear or contain fine eosinophilic material. They are cuboidal to polyhedral with round shrunken nuclei and prominent nucleoli [5]. Immunohistochemical study shows positive staining for CK7, EMA, and CAIX, and negative staining for CK20, CD10, PAX8, and calponin [5]. It is usually managed with surgical resection, with some cases treated with adjuvant radiotherapy. The lesions tend to have a good prognosis with no recurrence except for three cases, with one that showed a high-grade tumor with lymph node metastasis and recurrence with an expression of EGFR, one that was treated with endoscopic surgery and recurs after 35 months post-surgery, and another case that was associated with VHL syndrome [7-9].

VHL is an autosomal dominant disorder with high penetrance and variable expressivity. It is due to the inactivation of the VHL tumor suppressor gene on chromosome 3, which is a critical regularity chromosome that is responsible for stress adaptation through the VHL-HIF1A pathway [10]. Its downstream pathway includes carbonic anhydrase 9 (CAIX) and glucose transporter 1 (GLUT1). Many studies tried to investigate the association of CAIX and GLUT1 with tumors related to VHL syndrome. Chatzopoulos et al. studied the expression of CAIX and GLUT1 in VHL-related lesions and concluded that its expression was overexpressed in VHL-related lesions, which reflects the upregulation of the HIF1A pathway; thus, this combination helps in screening lesions of patients with VHL spectrum manifestations [11]. Additionally, another study stained many VHL-related paragangliomas with CAIX and found that membranous staining is a highly sensitive and specific marker for germline and somatic VHL mutations; however, the staining could be diffuse in all tumor cells or to a single cell in the whole section [12]. Additionally, Mete et al. studied the expression of inhibin and CAIX in paraganglioma and pheochromocytoma and its association with a pseudohypoxic state. They found that inhibin was associated with SDHx/ VHL-driven pseudohypoxia, with CAIX being the most specific marker for VHL-related pathogenesis marker [13]. Utilizing these markers, while not totally specific, could be helpful in countries with limited resources. Additionally, targeted therapy is being studied for this pathway.

It is not certain whether this lesion is associated with VHL. There are three cases of SNRCLA with VHL described in the literature [2,9,14]. All lesions were seen in patients in the fourth decade of life and were associated with non-specific symptoms. Additionally, all cases had a prior diagnosis of VHL-associated neoplasms and aggressive behavior, unlike our case, which was small in size with an indolent clinical course. Microscopically, two cases showed cystic proliferation of bland clear cells with distinct cell borders. In contrast, the other showed follicular proliferation of bland clear cells with distinct cytoplasmic borders and colloid-like secretion. Neither necrosis nor mitosis was identified. All cases stained positively with CK7 and EMA, while one stained positively with inhibin, which is similar to ours. All cases were negative for CD10, RCC, and PAX8 (Table 1). In our case, we do not think that it is associated with VHL syndrome as there is no history of other neoplasms like other cases. Unfortunately, we could not verify our suspicions as genetic testing for VHL was not available in our institute, as well as the surrogate markers for VHL (GLUT1, CAIX, and HIF1A).

**Table 1:** Comparison between our case and the cases reported in the literature that showed association with VHL syndrome VHL, von Hippel Lindau

Case	Xu et al., 2012 [14]	Cooper et al., 2017 [9]	Maharaj et al., 2022 [2]	Our case
Clinical	36-year-old female; non-specific symptoms; CNS neoplasms	35-year-old male; non-specific symptoms; CNS and renal neoplasms	35 years old male No- specific symptoms CNS and visceral neoplasms	41-year-old female; non-specific symptoms; no history of tumors
Histopathological	Microcytic proliferation; bland clear cells with distinct cell borders; no mitosis and necrosis	Tubulocystic proliferation; bland clear cells with brush borders; No mitosis	Follicular growth pattern with colloid-like material; bland clear cells with distinct cell borders; no mitosis and necrosis	Follicular to glandular; bland clear cells; no necrosis and low mitosis
Ancillary tests	Positive stains: CK7, EMA, and inhibin; negative stains: CD10, NSE, and S100	Positive stains: CK7 and EMA; negative stains: P63, inhibin, CD10, and RCC; KI67: low	Positive stains: CK7, PanCK, and S100; negative stains: CD10, RCC, and PAX8	Positive stains: CK7, EMA, and inhibin; negative stains: PAX8, RCC, CD10, and p63; KI67: low

Differential diagnoses of SNRCLA include metastatic carcinoma with clear cell changes, especially metastatic RCC. Patients should be investigated for other primary tumors from the kidney, especially since around 6% of RCC metastasize to the head and neck, with 20% of them to the sinonasal area [5]. Differentiation could be done based on full body scanning and histomorphology as clear cell renal cell carcinoma shows nuclear pleomorphism with abundant necrosis. On the other hand, SNRCLA is composed of bland cuboidal to clear columnar cells with minimum necrosis. Both could show increased vascularity and hemorrhage [5]. On immunohistochemistry, both show positivity for CAIX. At the same time, SNRCLA is negative for PAX8 and RCC and positive for CK7 [5]. Another differential diagnosis is clear cell carcinoma of the salivary gland, and it can be differentiated from SNRCLA by the absence of p63, p40, CK5/6, and lack of EWSR fusion that is usually positive in salivary gland tumor [15]. Other differentials include clear cell predominant mucoepidermoid carcinoma, squamous cell carcinoma with clear changes, and clear cell odontogenic carcinoma. Metastatic disease from the kidney and thyroid could be ruled out by a PET scan and negative RCC, PAX8, and TTF1 [16,17].

Finally, while we believe that our case is not associated with VHL syndrome, the unavailability of genetic testing and surrogate markers for VHL syndrome are major limitations of our study.

## Conclusions

SNRCLA can pose a diagnostic challenge because it is a rare neoplasm with no specific clinical or radiological findings, with multiple histopathological differential diagnoses. Additionally, although we do not think that our case is related to VHL syndrome, some cases are associated with VHL; hence, testing patients for possible VHL syndrome is recommended.
